# Potential Novel Biomarkers of Obstructive Nephropathy in Children with Hydronephrosis

**DOI:** 10.1155/2018/1015726

**Published:** 2018-09-13

**Authors:** Beata Bieniaś, Przemysław Sikora

**Affiliations:** Department of Pediatric Nephrology, Medical University of Lublin, Gębali 6, 20-093 Lublin, Poland

## Abstract

Obstructive nephropathy (ON) secondary to the congenital hydronephrosis (HN) is one of the most common causes of chronic kidney disease in children. Neither currently used imaging techniques nor conventional laboratory parameters are sufficient to assess the onset and outcome of this condition; hence, there is a need to prove the usefulness of newly discovered biomarkers of kidney injury in this respect. The purpose of the study was to assess the urinary excretion of alpha-GST, pi-GST, NGAL, and KIM-1 and the serum level of NGAL in children with congenital unilateral hydronephrosis secondary to ureteropelvic junction obstruction. The results were evaluated in relation to severity of HN, the presence of ON, relative function of an obstructed kidney, and the presence of proteinuria. The study comprised 45 children with HN of different grades and 21 healthy controls. Urinary and serum concentrations of biomarkers were measured using specific ELISA kits. Urinary biomarker excretions were expressed as a biomarker/creatinine (Cr) ratio. Patients with the highest grades of HN showed significantly increased values of all measured biomarkers, whereas those with the lowest grades of HN displayed only significant elevation of urinary alpha-GST and the serum NGAL. Urinary NGAL positively correlated with percentage loss of relative function of an obstructed kidney in renal scintigraphy. In patients with proteinuria, significantly higher urinary alpha-GST excretion was revealed as compared to those without this symptom. The ROC curve analysis showed the best diagnostic profile for urinary alpha-GST/Cr and NGAL/Cr ratios in the detection of ON. In conclusion, the results of the study showed that urinary alpha-GST and NGAL are promising biomarkers of ON. Ambiguous results of the remaining biomarkers, i.e., urinary pi-GST and KIM-1, and serum NGAL level may be related to a relatively small study group. Their utility in an early diagnosis of ON should be reevaluated.

## 1. Introduction

Obstructive nephropathy (ON) is a chronic inflammatory process characterized by renal scarring resulting from obstructive uropathy (hydronephrosis). Scarring of an obstructed kidney may lead to impairment of its function.

ON secondary to the congenital hydronephrosis (HN) is one of the most common causes of chronic kidney disease (CKD) in children [1–3]. Ureteropelvic junction obstruction (UPJO) has been revealed as the main cause of significant HN [3].

Etiopathogenesis of ON is complex, but the primary and secondary injuries to the renal tubular epithelial cells are believed to be especially important [4]. They lead to tubulointerstitial inflammation, tubular atrophy, and fibrosis. Unfortunately, neither currently used imaging techniques nor conventional laboratory parameters are sufficient to assess the onset and outcome of this condition. In the recent years, several biomarkers of tubulointerstitial fibrosis have been discovered and studied in different renal diseases. Some of them like neutrophil gelatinase-associated lipocalin (NGAL) and kidney injury molecule-1 (KIM-1) have been tested with uncertain results in patients with ON, whereas other biomarkers like glutathione S-transferases (GSTs) are still waiting for evaluation.

To provide a new insight into this issue, we studied the usefulness of GSTs, NGAL, and KIM-1 as potential biomarkers of ON.

## 2. Purpose of the Study

The purpose of the study was to assess the urinary excretion of alpha-GST, pi-GST, NGAL, and KIM-1 and the serum level of NGAL in children with congenital unilateral hydronephrosis secondary to UPJO. These biomarkers were evaluated in relation to severity of HN, the presence of ON, relative function of an obstructed kidney, and the presence of proteinuria.

## 3. Patients, Material, and Methods

Baseline characteristics of patients and controls are presented in Table 1. The study comprised 45 children (31 boys and 14 girls) aged 2–17 years (median = 11.0 years) with congenital unilateral HN due to UPJO diagnosed and treated in the Department of Pediatric Nephrology, Children's University Hospital in Lublin, Poland. In 25 children, the HN was diagnosed prenatally. The patients were divided into three subgroups A–C according to the Onen HN ultrasound grading system [5] as follows: stage 1—dilatation of renal pelvis alone, stage 2—like stage 1 plus caliceal dilatation, stage 3—like stage 2 plus <1/2 (mild-to-moderate) renal parenchymal loss, and stage 4—like stage 3 plus >1/2 (severe) renal parenchymal loss (cyst-like kidney with no visually significant renal parenchyma). 25/45 (55.6%) children with HN grades 3 and 4 were classified into the group A, 11/45 (24.4%) with HN grade 2 into the group B, and 9/45 (20%) with HN grade 1 into the group C. To detect ON defined as renal parenchymal defects with decreased relative function of an obstructed kidney, a dynamic renal scintigraphy using technetium-99m-L,L-ethylenedicysteine was performed. 28/45 (62.2%) patients predominantly from the group A and group B (21 and 7, respectively) showed features of ON with impaired relative function of an obstructed kidney from 15 to 35%.

10/45 (22.2%) patients from the group A, including 7 with ON, disclosed pathological proteinuria (urinary protein/creatinine ratio: 0.21–0.4 mg/mg, median: 0.24 mg/mg).

All children had normal estimated glomerular filtration rate (eGFR) > 90 ml/min/1.73 m^2^ calculated by the Schwartz formula: 0.55 × body height (cm)/serum creatinine level (mg/dl) [6]. The age- and sex-matched 21 healthy children were controls. They were referred to the outpatient clinic, Children's University Hospital, with suspicion of renal diseases that were excluded.

To evaluate the designed laboratory parameters, the midstream first morning urine and serum samples were collected from each study participant on the same day. Serum and urinary creatinine concentrations were determined by Jaffe's test. Standard laboratory techniques were used to assess the magnitude of proteinuria.

Urinary alpha-GST (USCNK, China), urinary pi-GST (Immundiagnostik AG, Germany), urinary KIM-1, and urinary and serum NGAL (USCNK, China) concentrations were measured using specific enzyme-linked immunosorbent assay (ELISA) kits after prior preparation of urine and serum samples following the manufacturer's instructions.

The urinary biomarker excretions were expressed as a biomarker/creatinine ratio in nanograms per milligram of creatinine (ng/mg Cr). The urinary protein excretion was showed as a protein/creatinine ratio in milligram per milligram of creatinine (mg/mg Cr).

The differences of urinary alpha-GST, urinary pi-GST, urinary KIM-1, and urinary and serum NGAL between groups of patients with ON and patients with HN without ON were assessed. Additionally, the comparison of measured biomarkers in groups of patients with proteinuria and patients with HN without proteinuria was performed.

The statistical analysis was performed using STATISTICA 12.5. Differences between groups were assessed using a nonparametric Mann-Whitney *U* test, and correlation coefficients were calculated using a Spearman test. *p* value ≤ 0.05 was considered significant.

The evaluation of clinical utility and significance of measured parameters as biomarkers of ON was performed by ROC curve analyses.

## 4. Ethics Statement

The study was approved by Ethics Committee of the Medical University of Lublin.

Informed consent was obtained from all individual participants included in the study, either the patients or the parents or legal guardians.

## 5. Results

### 5.1. Biomarker Measurements

The results of assessed biomarkers in the study groups as compared to controls are presented in Tables 2–4.

In the group A, median urinary alpha-GST/Cr, pi-GST/Cr, NGAL/Cr, and KIM-1/Cr ratios and the serum NGAL level were significantly higher than those in controls (*p* < 0.05) (Table 2).

In the group B, median urinary alpha-GST/Cr and KIM-1/Cr ratios and the serum NGAL level were significantly higher in comparison with controls (*p* < 0.05). No significant differences of urinary pi-GST/Cr and NGAL/Cr ratios between patients from the group B and controls were observed (Table 3).

In the group C, significant elevation of the median urinary alpha-GST/Cr ratio and serum NGAL level was found (*p* < 0.05). In comparison with controls, there were no significant differences of urinary pi-GST/Cr, NGAL/Cr, and KIM/Cr ratios (Table 4).

**Figure 1:**
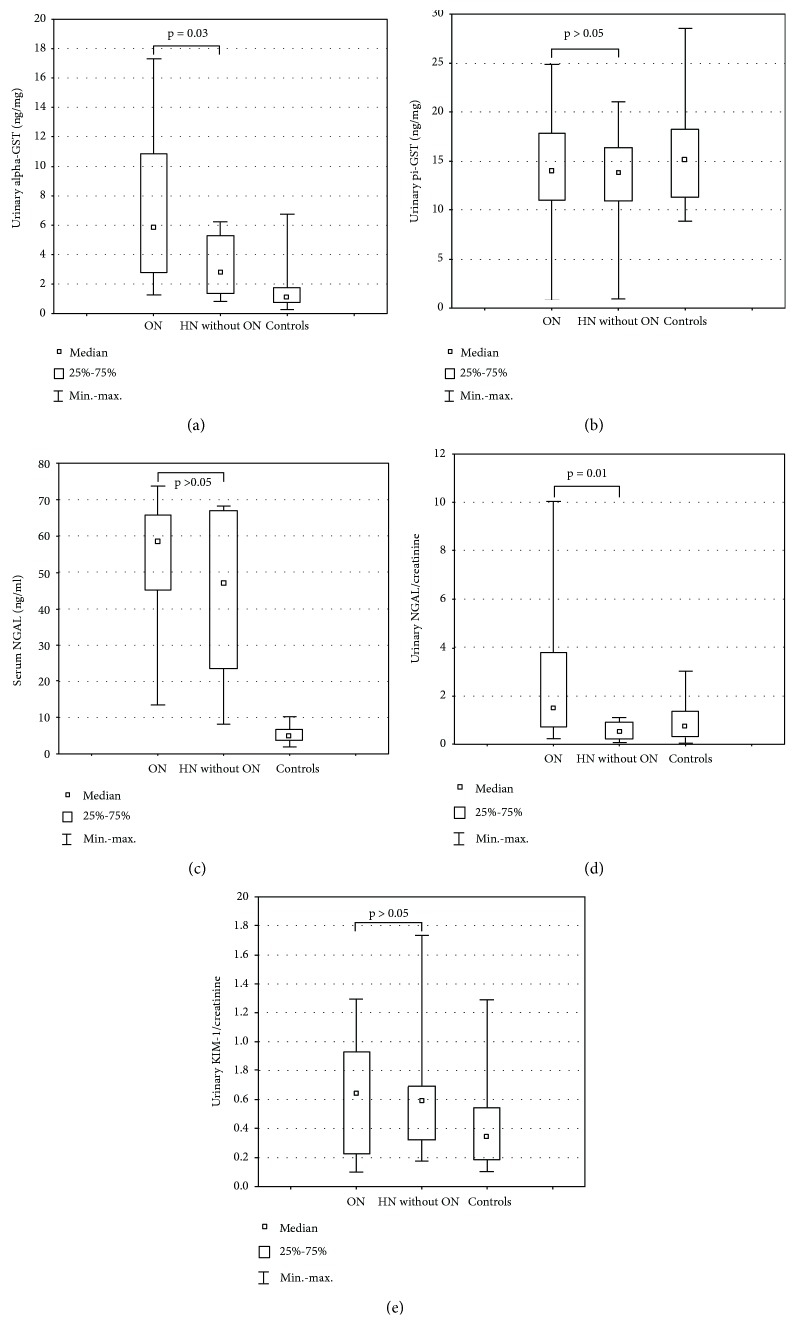
Urinary alpha-GST (a), pi-GST (b), NGAL (c, d), and KIM-1 (e) ratios in patients with obstructive nephropathy (ON), patients with hydronephrosis without obstructive nephropathy (HN without ON), and controls.

Patients with ON had significantly increased median urinary alpha-GST/Cr and NGAL/Cr ratios in comparison with those without ON (*p* = 0.03 and *p* = 0.01, respectively) (Figure 1). There were no significant differences of urinary pi-GST/Cr and KIM-1/Cr ratios and the serum NGAL level.

**Figure 2:**
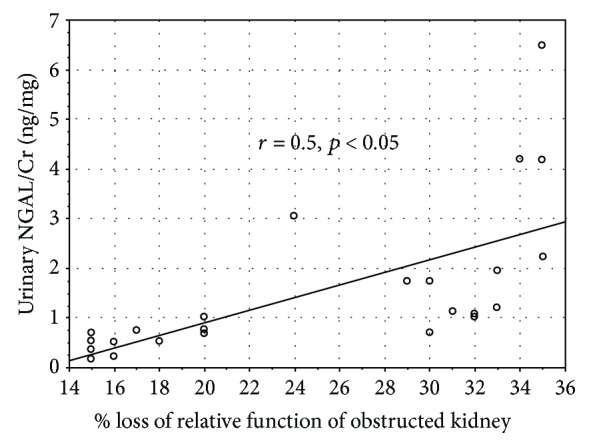
Correlation between the urinary NGAL/Cr ratio and percentage loss of relative function of an obstructed kidney in dynamic renal scintigraphy.

In addition, there was positive correlation between the urinary NGAL/Cr ratio and percentage loss of relative function of an obstructed kidney (*r* = 0.5, *p* < 0.05) (Figure 2). The latter did not significantly correlate with urinary alpha-GST/Cr, pi-GST/Cr, and KIM-1/Cr ratios and the serum NGAL level.

**Figure 3:**
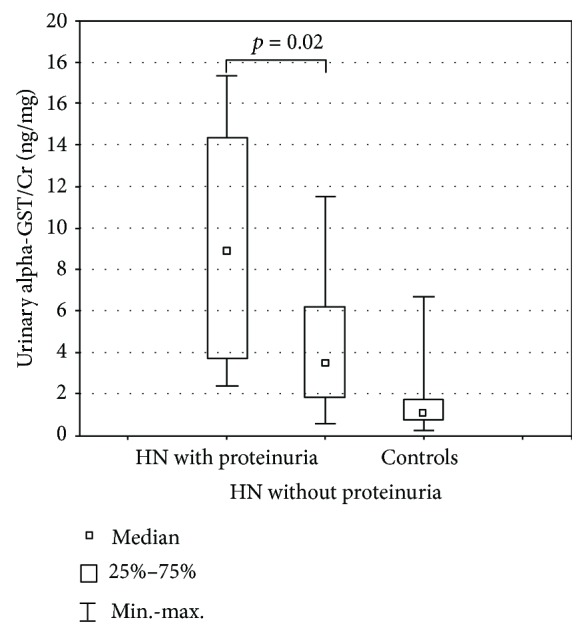
Urinary alpha-GST/Cr ratio in hydronephrotic patients (HN) with and without proteinuria.

In patients with proteinuria, only the median urinary alpha-GST/Cr ratio was significantly higher as compared to those without this symptom (*p* = 0.02) (Figure 3).

### 5.2. ROC Analysis

**Figure 4:**
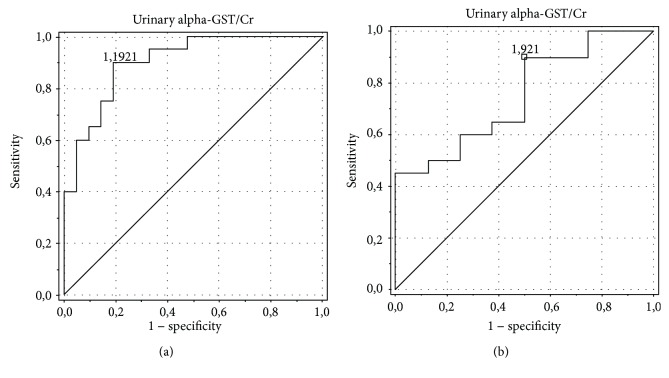
ROC analysis for the urinary alpha-GST/Cr ratio in the detection of obstructive nephropathy (ON). (a) Patients with obstructive nephropathy (ON) vs. controls. AUC of 0.902, an optimal cut-off value of 0.046 ng/mg Cr, sensitivity of 81.8%, specificity of 84.6%. (b) Patients with obstructive nephropathy (ON) vs. patients with hydronephrosis without obstructive nephropathy (HN without ON). AUC of 0.750, an optimal cut-off value of 0.098 ng/mg Cr, sensitivity of 84.6%, specificity of 69.2%.

**Figure 5:**
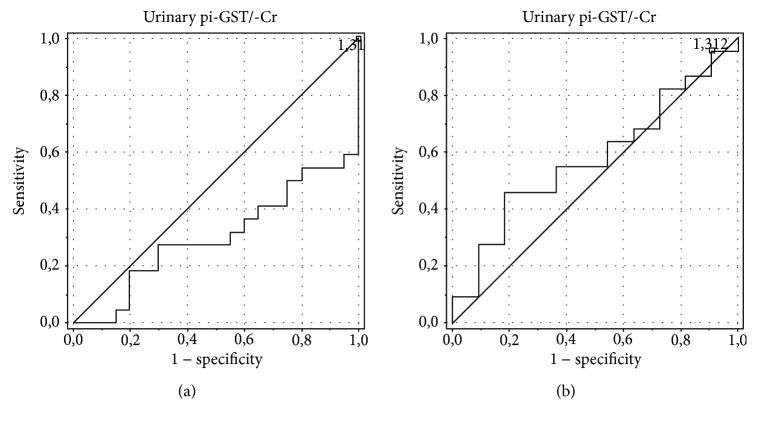
ROC analysis for the urinary pi-GST/Cr ratio in the detection of obstructive nephropathy (ON). (a) Patients with obstructive nephropathy (ON) vs. controls. AUC of 0.3, an optimal cut-off value of 0.082 ng/mg Cr, sensitivity of 92.3%, specificity of <1%. (b) Patients with obstructive nephropathy (ON) vs. patients with hydronephrosis without obstructive nephropathy (HN without ON). AUC of 0.574, an optimal cut-off value of 0.103 ng/mg Cr, sensitivity of 92.3%, specificity of 7.7%.

**Figure 6:**
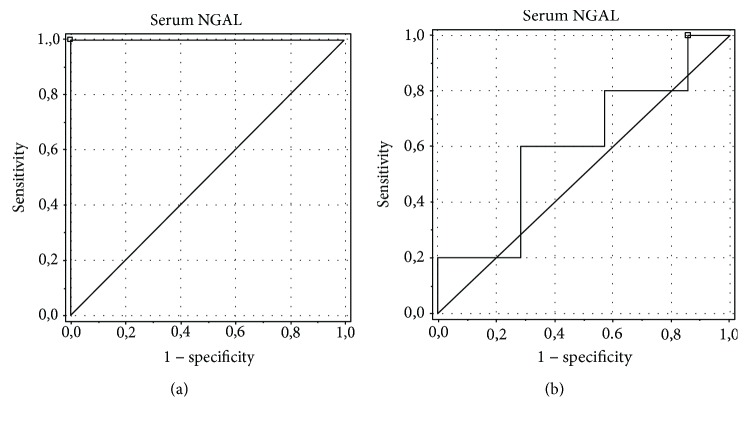
ROC analysis for the serum NGAL level in the detection of obstructive nephropathy (ON). (a) Patients with obstructive nephropathy (ON) vs. controls. AUC of 1.0, an optimal cut-off value of 0.0 ng/ml, sensitivity of 100%, specificity of 100%. (b) Patients with obstructive nephropathy (ON) vs. patients with hydronephrosis without obstructive nephropathy (HN without ON). AUC of 0.6, an optimal cut-off value of 0.145 ng/ml, sensitivity of 100%, specificity of 12.5%.

**Figure 7:**
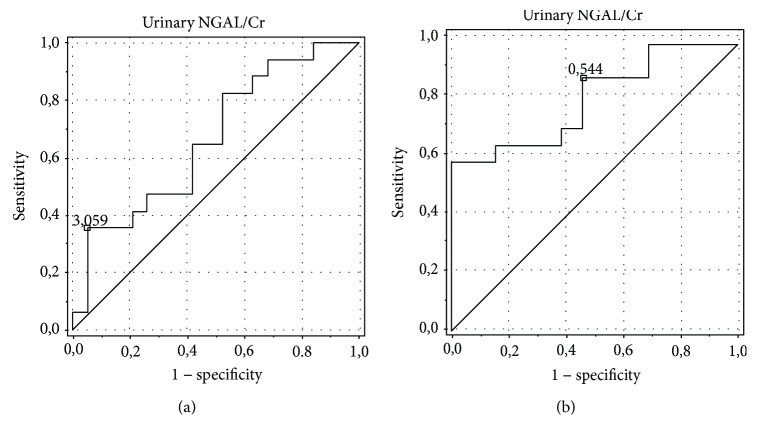
ROC analysis for the urinary NGAL/Cr ratio in the detection of obstructive nephropathy (ON). (a) Patients with obstructive nephropathy (ON) vs. controls. AUC of 0.663, an optimal cut-off value of 0.091 ng/mg Cr, sensitivity of 39.3%, specificity of 58.3%. (b) Patients with obstructive nephropathy (ON) vs. patients with hydronephrosis without obstructive nephropathy (HN without ON). AUC of 0.805, an optimal cut-off value of 0.079 ng/mg Cr, sensitivity of 78.6%, specificity of 58.3%.

**Figure 8:**
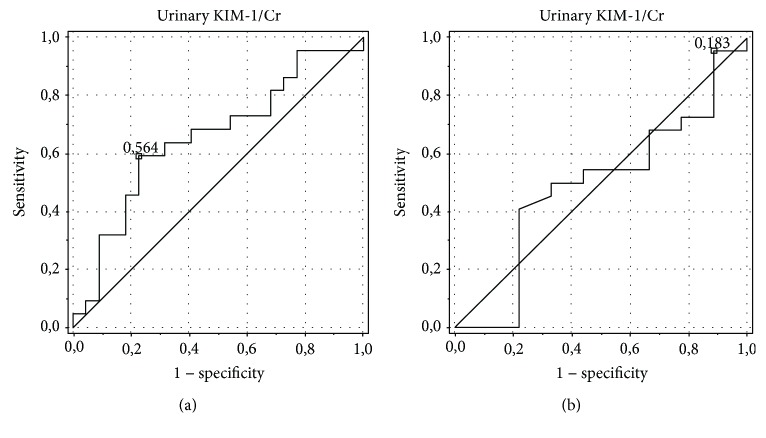
ROC analysis for the urinary KIM-1/Cr ratio in the detection of obstructive nephropathy (ON). (a) Patients with obstructive nephropathy (ON) vs. controls. AUC of 0.653, an optimal cut-off value of 0.084 ng/mg Cr, sensitivity of 55.6%, specificity of 69.6%. (b) Patients with obstructive nephropathy (ON) vs. patients with hydronephrosis without obstructive nephropathy (HN without ON). AUC of 0.487, an optimal cut-off value of 0.119 ng/mg Cr, sensitivity of 88.9%, specificity of 16.7%.

The analysis showed the best diagnostic profiles in the detection of ON for the urinary alpha-GST/Cr ratio (area under the curve (AUC) of 0.75, an optimal cut-off value of 0.098 ng/mg with sensitivity and specificity of 84.6% and 69.2%, respectively) and urinary NGAL/Cr ratio (AUC of 0.805, an optimal cut-off value of 0.08 ng/mg with sensitivity and specificity of 78.6% and 58.3%, respectively). AUC for the serum NGAL level was 0.6, with an optimal cut-off value of 0.145 ng/ml, sensitivity of 100%, and specificity of 12.5%. The urinary pi-GST/Cr ratio was characterized by AUC of 0.574, an optimal cut-off value of 0.103 ng/mg Cr, sensitivity of 92.3%, and specificity of 7.7%, and the urinary KIM-1/Cr ratio was characterized by AUC of 0.487, an optimal cut-off value of 0.119 ng/mg Cr, sensitivity of 88.9%, and specificity of 16.7% (Figures 4–8).

## 6. Discussion

GST is a cytosolic enzyme. Its isoforms alpha and pi (alpha-GST, pi-GST) are typical of a human kidney [7]. The alpha-GST is expressed in proximal tubular epithelial cells, whereas the pi-GST is a characteristic of distal tubular epithelial cells. Both isoforms of GST are excessively released from injured tubular epithelial cells into the urine, and they recently were proposed as promising biomarkers of tubulointerstitial fibrosis in patients with proteinuric kidney disease [8, 9]. To the best of our knowledge, in patients with HN, no studies on urinary GST excretion and their usefulness as biomarkers of ON have been reported so far. In our study, all patients with HN were characterized by significantly higher urinary alpha-GST excretion in comparison with controls. In addition, urinary alpha-GST excretion was significantly higher in children with HN and ON as compared to those without ON. Similarly, our patients with proteinuria displayed significantly higher urinary alpha-GST excretion than those without this symptom. The ROC curve analysis showed a very good diagnostic profile for the urinary alpha-GST/Cr ratio in the detection of ON.

In our study, urinary pi-GST excretion was significantly higher in patients with grades 3 and 4 HN as compared to controls but the ROC curve analysis did not confirm clinical utility of the urinary pi-GST/Cr ratio in the detection of ON.

NGAL is considered to be another sensitive and early biomarker of tubulointerstitial fibrosis. It is a small (25 kD) protein, locally synthesized in renal tubular epithelial cells [10] and released into urine. Initially, NGAL was thought to be a biomarker of acute kidney injury (AKI) [11] but recent studies demonstrated its increased urinary excretion also in patients with various chronic nephropathies. Furthermore, correlation between urinary NGAL excretion and severity of local inflammation and kidney function was observed [12–15]. NGAL may also be released into the circulation from damaged renal tubular epithelial cells. Its elevated serum level is suggested to be a risk factor for CKD progression [16–19]. In our study, it was also showed that urinary NGAL excretion may be a potential biomarker of ON in patients with HN due to UPJO [18–22]. We found that urinary NGAL excretion was significantly higher in patients with HN and ON as compared to patients with HN and without ON. In our study, urinary NGAL excretion positively correlated with a deterioration of relative function of an obstructed kidney in dynamic renal scintigraphy. Moreover, similar to the urinary alpha-GST/Cr ratio, the ROC curve analysis showed a very good diagnostic profile for the urinary NGAL/Cr ratio in the detection of ON. Interestingly, Gerber et al. [23] did not find significant differences in urinary NGAL excretion between patients with UPJO and controls. Nevertheless, their results might be influenced by relatively small number of cases.

In our study, in contrast to urinary NGAL excretion, the serum NGAL level was significantly higher in all hydronephrotic children in comparison with controls. Unfortunately, in the ROC curve analysis, a diagnostic profile for the serum NGAL level in the detection of ON was not so good as those for urinary NGAL/Cr and urinary alpha-GST/Cr ratios.

Transmembrane renal tubular epithelial cell glycoprotein KIM-1 is the most recently recognized biomarker of tubulointerstitial fibrosis. Its physiological role is still unclear, but it is markedly upregulated in proximal tubular epithelial cells in experimental and clinical conditions associated with kidney damage [24]. Its elevated urinary excretion was observed in both AKI [25, 26] and various chronic kidney diseases [12, 27, 28]. KIM-1 was also suggested to be an indicator of the conversion of AKI to CKD [29]. Vaidya et al. [28] showed that urinary KIM-1 excretion reflected better severity of tubulointerstitial fibrosis than a traditional biomarker—N-acetyl-*β*-D-glucosaminidase.

The usefulness of urinary KIM-1 excretion as a new early biomarker of ON in patients with HN was reported by several recent studies [21, 22, 25, 29, 30]. However, in the study by Noyan et al. [20], hydronephrotic children with kidney dysfunction were not characterized by increased urinary KIM-1 excretion. Similarly, Gerber et al. [23] did not observe elevated urinary KIM-1 excretion in patients with UPJO. In our study, significantly higher urinary KIM-1 excretion was noted in patients with grades 2–4 HN but not in those with ON and in those with proteinuria. The ROC curve analysis did not show a sufficient diagnostic profile for the urinary KIM-1/Cr ratio in the detection of ON.

## 7. Conclusions

Our results suggest that urinary alpha-GST and NGAL are promising biomarkers of ON. It is conceivable that ambiguous results regarding the remaining biomarkers, i.e., urinary pi-GST and KIM-1, and serum NGAL level may be related to a relatively small study group. Therefore, their utility in an early diagnosis of ON should be reevaluated by more extended investigations.

## Figures and Tables

**Table 1:** Characteristics of study and control groups.

Parameter	Study group median (range) & number of patients	Control group
Number of patients	45	21
Gender (male/female)	31/14	16/5
Age (years)	11.0 (2–17)	12.3 (3–17)
GFR (ml/min/1.73 m^2^)	126.3 (97–162)	139 (102–145)
Number of patients in groups (A/B/C)	25/11/9	—
Number of patients with obstructive nephropathy	28/45 (62.2%)	—
Number of patients with proteinuria	10/45 (22.2%)	—
Protein/creatinine ratio (mg/mg)	0.24 (0.21–0.4)	0.09 (0–0.15)

**Table 2:** The results of the urinary excretion of alpha-GST/Cr, pi-GST/Cr, NGAL/Cr, and KIM-1/Cr and serum NGAL levels in the study group A and control group.

	Study group A median (range)	Control group median (range)	Statistical analysis
Alpha-GST/Cr (ng/mg)	4.51 (0.54–17.3)	1.11 (0.26–3.5)	*p* = 0.0001
pi-GST/Cr (ng/mg)	30.4 (17.5–24.9)	14.6 (7.4–28.5)	*p* = 0.03
uNGAL/Cr (ng/mg)	1.73 (0.17–10.0)	0.83 (0.04–9.5)	*p* = 0.02
sNGAL (ng/ml)	59.9 (45.2–85.3)	4.8 (2.1–10.4)	*p* = 0.00003
KIM-1/Cr (ng/mg)	2.4 (0.2–5.1)	0.28 (0.06–1.06)	*p* = 0.02

uNGAL: urinary NGAL; sNGAL: serum NGAL; Cr: creatinine.

**Table 3:** The results of the urinary excretion of alpha-GST/Cr, pi-GST/Cr, NGAL/Cr, and KIM-1/Cr and serum NGAL levels in the study group B and control group.

	Study group B median (range)	Control group median (range)	Statistical analysis
Alpha-GST/Cr (ng/mg)	6.17 (0.8–11.74)	1.11 (0.26–3.5)	*p* = 0.008
pi-GST/Cr (ng/mg)	17.9 (7.8–20.83)	14.6 (7.4–28.5)	NS
uNGAL/Cr (ng/mg)	1.41 (0.3–5.2)	0.83 (0.04–9.5)	NS
sNGAL (ng/ml)	58.5 (13.4–73.8)	4.8 (2.1–10.4)	*p* = 0.001
KIM-1/Cr (ng/mg)	0.58 (0.2–0.96)	0.28 (0.06–1.06)	*p* = 0.02

uNGAL: urinary NGAL; sNGAL: serum NGAL; Cr: creatinine.

**Table 4:** The results of the urinary excretion of alpha-GST/Cr, pi-GST/Cr, NGAL/Cr, and KIM-1/Cr and serum NGAL levels in the study group C and control group.

	Study group C median (range)	Control group median (range)	Statistical analysis
Alpha-GST/Cr (ng/mg)	4.73 (0.81–11.7)	1.11 (0.26–3.5)	*p* = 0.008
pi-GST/Cr (ng/mg)	16.3 (7.6–23.1)	14.6 (7.4–28.5)	NS
uNGAL/Cr (ng/mg)	0.51 (0.07–9.7)	0.83 (0.04–9.5)	NS
sNGAL (ng/ml)	33.6 (8.3–68.4)	4.8 (2.1–10.4)	*p* = 0.001
KIM-1/Cr (ng/mg)	0.68 (0.2–1.3)	0.28 (0.06–1.06)	NS

uNGAL: urinary NGAL; sNGAL: serum NGAL; Cr: creatinine.

## Data Availability

The data used to support the findings of this study are available from the corresponding author upon request.
